# XGBoost-Based Framework for Smoking-Induced Noncommunicable Disease Prediction

**DOI:** 10.3390/ijerph17186513

**Published:** 2020-09-07

**Authors:** Khishigsuren Davagdorj, Van Huy Pham, Nipon Theera-Umpon, Keun Ho Ryu

**Affiliations:** 1 Database and Bioinformatics Laboratory, College of Electrical and Computer Engineering, Chungbuk National University, Cheongju 28644, Korea; khishigsurend@gmail.com; 2 Faculty of Information Technology, Ton Duc Thang University, Ho Chi Minh 700000, Vietnam; phamvanhuy@tdtu.edu.vn; 3 Department of Electrical Engineering, Faculty of Engineering, Chiang Mai University, Chiang Mai 50200, Thailand; nipon.t@cmu.ac.th; 4 Biomedical Engineering Institute, Chiang Mai University, Chiang Mai 50200, Thailand

**Keywords:** smoking, noncommunicable disease, feature selection, extreme gradient boosting

## Abstract

Smoking-induced noncommunicable diseases (SiNCDs) have become a significant threat to public health and cause of death globally. In the last decade, numerous studies have been proposed using artificial intelligence techniques to predict the risk of developing SiNCDs. However, determining the most significant features and developing interpretable models are rather challenging in such systems. In this study, we propose an efficient extreme gradient boosting (XGBoost) based framework incorporated with the hybrid feature selection (HFS) method for SiNCDs prediction among the general population in South Korea and the United States. Initially, HFS is performed in three stages: (I) significant features are selected by t-test and chi-square test; (II) multicollinearity analysis serves to obtain dissimilar features; (III) final selection of best representative features is done based on least absolute shrinkage and selection operator (LASSO). Then, selected features are fed into the XGBoost predictive model. The experimental results show that our proposed model outperforms several existing baseline models. In addition, the proposed model also provides important features in order to enhance the interpretability of the SiNCDs prediction model. Consequently, the XGBoost based framework is expected to contribute for early diagnosis and prevention of the SiNCDs in public health concerns.

## 1. Introduction

Noncommunicable diseases (NCDs) have emerged as a major public health problem in the world. About 40 million people die from NCDs each year, equivalent to 70% of all deaths globally. The major risk factors of developing NCDs consist of tobacco use, physical inactivity, alcohol use, and unhealthy diets [1]. Approximately 80% of all heart disease, stroke, and diabetes would be prevented if these major risk factors were eliminated. Tobacco use is negatively associated with all of the United Nation (UN)’s Sustainable Development Goals (SDGs). In particular, smoking cessation plays an extensive role in a global effort to achieve the SDGs target to reduce deaths from NCDs by one-third by 2030 [2,3]. Studies by public health experts found that smokers were more likely to become infected with outbreaks of Middle East respiratory syndrome coronavirus (MERS-CoV) and corona virus disease (COVID) 19 compared to non-smokers [4,5].

The World Health Organization (WHO) introduced the MPOWER package, corresponding to assist in country-level implementation of interventions to increase smoking cessation, as ratified by the WHO Framework Convention on Tobacco Control (WHO-FCTC). The MPOWER package is part of the WHO action plan for the prevention and control of noncommunicable diseases. South Korea has ratified the WHO-FCTC and, since 2005, has launched anti-smoking clinics at community health centers across the country. However, the prevalence of NCDs continues to remain high, accounting for 81% of all deaths and seven of the top causes of death in South Korea [6]. In recent years, the United States government has aimed to expand its role in addressing the challenges of SiNCDs. NCDs account for 89% of all deaths in the United States, which far exceeds the cases of infectious diseases as considered causes of death [7]. Current smokers who suffer from NCDs need lifelong treatment. One of the main causes of this adverse scenario is the fact that smoking-induced noncommunicable diseases (SiNCDs) is mostly diagnosed in late stages.

Nowadays, predictive models are frequently employed in early diagnosis and forecasting of smoking-associated illnesses and diseases [8,9,10,11,12]. Early detection and efficient treatments are solutions for reducing death rates caused by chronic diseases. In one study [13], the authors studied the association between environmental factors and the development of Crohn’s disease among Japanese. Their results suggest that passive smoking history is associated with the development of Crohn’s disease. Another study [14] evaluated the association between Parkinson’s disease and rural living, farming, pesticide use, and cigarette smoking. The weight of the evidence and meta-analysis showed that there is a causal relationship between the risk of Parkinson’s disease and cigarette smoking, which has been consistently discovered in related literature. In contrast, rural living, well-water consumption, farming and the use of pesticides, herbicides, insecticides, and fungicides were less consistent within Parkinson’s disease. Furthermore, one study [15] focused on the role of feature risk pathways in smoking-induced lung cancer using patient data from the Gene Expression Omnibus database. They optimized the feature sets using the anomaly score and the recursive feature elimination (RFE) method. Then, the support vector machine (SVM) based prediction model was used. Their study concluded that smoking is the main cause of lung cancer; moreover, stress and self-protection mechanisms in a living organism can be identified as complex factors. In another study [16], authors developed an automatic classifier to increase the accuracy of the forced oscillation technique for early diagnosis of smoking-induced respiratory changes. They utilized several machine learning techniques, such as logistic linear classifiers, k nearest neighbor (KNN), neural networks (NN), and SVM. As their result, KNN and SVM classifiers resulted in a further increase in diagnostic accuracy.

Artificial intelligence (AI) utilization of automated diagnosis processes can highlight enhanced rules in the decision support system regarding patient health care. However, there are some difficulties in the selection of representative features and suitable classifier. Numerical studies have proposed selecting features using information gain, gain ratio, and correlation coefficients. However, these techniques do not consider the interactions among the features, and are not suitable for direct application to ensemble generation [17]. Moreover, it is evidently seen that ensemble-based classifiers improve the performance better than that of any single classifier [18]. Otherwise, a sequential ensemble learns to generate a model, and one tries to reduce the bias of the combined estimator and reach close to actual predictions. Thus, new models are learned from the mistakes of previous models by boosting techniques. Tree boosting provides high performance clinical predictive modeling; furthermore, it allows representation of feature importance and ranking [19].

Therefore, we propose efficient extreme gradient boosting (XGBoost) based framework incorporated with the hybrid feature selection (HFS) method for SiNCDs prediction, using real-world National Health and Nutrition Examination Survey (NHANES) datasets of South Korea and the United States. Firstly, HFS is performed in three stages: (I) significant features are selected based on statistical hypothesis tests, such as t-test and chi-square; (II) multicollinearity analysis serves to obtain dissimilar features; followed by (III) final selection of best representative features is done based on least absolute shrinkage and selection operator (LASSO). Then, selected features are fed into the XGBoost predictive model. Finally, our proposed model provides feature importance score based on XGBoost. Therefore, the proposed model is compared and contrasted against several existing baseline models, such as logistic regression (LR), random forest (RF), KNN, multilayer perceptron (MLP), NN, support vector machine recursive feature elimination (SVM-RFE), and RF feature importance (RFFI) feature selection methods. The accuracy, sensitivity, specificity, precision, f-scores, and area under the curve (AUC) analysis are employed to evaluate model performances. The main contributions of this study are as follows:

- Proposing an efficient extreme gradient boosting (XGBoost) based framework incorporated with the hybrid feature selection (HFS) method for smoking–induced noncommunicable diseases (SiNCDs) prediction.
- Applying the XGBoost based framework to real-world NHANES datasets of South Korea and the United States. Our empirical comparison analysis shows that the proposed model outperformed existing baseline models.
- Findings are expected to contribute toward achieving good health and wellbeing (ultimate targets of SDGs of UN).

The remainder of this paper is logically structured as follows: Section 2 introduces the proposed framework—elaborating on its two main components: the three-stage HFS method and a brief introduction of the XGBoost algorithm. Therefore, it includes the experimental setup, regularizing hyperparameters. Section 3 provides discussed datasets, baseline models, and overall experimental results. Finally, the study is concluded in Section 4.

## 2. Materials Methods

### 2.1. Proposed Framework

**Figure 1:**
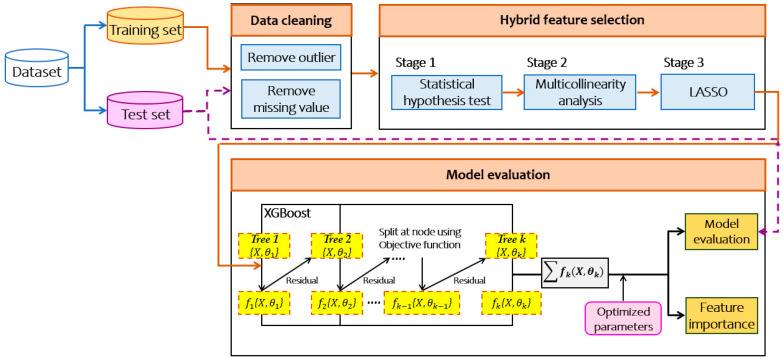
Extreme gradient boosting (XGBoost) based framework for smoking-induced noncommunicable diseases prediction. LASSO: least absolute shrinkage and selection operator.

In this paper, we propose an efficient extreme gradient boosting (XGBoost) based framework incorporated with the hybrid feature selection (HFS) method for smoking-induced noncommunicable diseases prediction. From its illustration in Figure 1, the proposed framework comprises of three main components: first, three-stage HFS, and then application of XGBoost to build the model. Finally, it provides the feature importance scores.

#### 2.1.1. Three-Stage HFS

The feature selection to reduce the data dimensionality and keep only the important features is performed in three stages.

- Step 1: Statistical Hypothesis Test (*t*-test and *p*-value)
  The first stage excludes redundant and irrelevant features in order to reduce the complexity for training model. For this purpose, it assesses chi-square test for categorical features and *t*-test for continuous features to accept or reject the alternative hypothesis. After the test, if deemed significant, features are stored for the first stage filtering. Otherwise, such features are excluded.
- Step 2: Multicollinearity Analysis
  The key assumption behind the multicollinearity analysis [20] is that it indicates the correlation between independent features. The value of variance inflation factor is used to verify multicollinearity in regression analysis. In essence, this step takes the complete set of features and loops through all of them applying the appropriate test.
- Step 3: Least Absolute Shrinkage and Selection Operator (LASSO)
  LASSO [21] has been extensively used in both fields of statistics and machine learning. Several studies [22,23] proposed the LASSO method for estimating the causal effect to identify their outcomes. A rational decision is taken to execute the LASSO in order to select a group of features simultaneously for a given task. During the feature selection process, LASSO penalizes the coefficients of the regression features, regularizing some of them to zero. On the contrary, features that still have a non-zero coefficient after the regularizing process remain to be part of the training model. This stage allows us to prevent the predictive of the causal inference problem.

#### 2.1.2. XGBoost Classifier

XGBoost is an efficient and scalable machine learning classifier, which was popularized by Chen and Guestrin in 2016 [24]. Gradient boosting decision tree is the original model of XGBoost, which combines multiple decision trees in boosting way. In general, each new tree is created to reduce the residual of the previous model by the gradient boosting. Residual is designated by the differences between the actual and predicted values. Until the number of decision trees specify threshold, the model has been trained. XGBoost has following the same principle of gradient boosting; it uses the number of boosts, learning rate, subsampling ratio, and maximum tree depth to control overfitting and enhance the better performance. More importantly, XGBoost optimizes the objective of function, size of the tree, and magnitude of the weights, which are controlled by standard regularization parameters. The XGBoost accomplishes superior performance with numerous hyperparameters in specific searching space as summarized in Table 1.

According to the hyperparameters, gamma γ∈(0,+∞) denotes minimum loss reduction, which requires to make a split for making the partition on a leaf node of the tree. Minimum child weight $w_{mc}\in \left(0,+\infty \right)$ defines as minimum sum of instance weight, which means if the tree partition step results in a leaf node with the sum of instance weight less than $w_{mc},$ then the tree will discard further partition. Early stop algorithm works for finding the optimal epoch number referring to given other hyperparameters. Finally, XGBoost also offered subsampling techniques and $r_{c}\;\in \left(0,\;1\right)$ column subsample ratio constructs in each tree. In the final step, grid search is used to regulate the hyperparameters in order to minimize the classification error.

### 2.2. Experimental Setup

#### 2.2.1. Experimental Environment

In this study, all experiments were performed on computer with 3.20 GHz, Intel Core i5-8250U (Intel Corporation, Santa Clara, CA, USA), and 8 GB Random access memory (RAM) using a Microsoft Windows 10 operating system (Microsoft Corporation, Redmond, WA, USA). Scikit-learn, Statsmodels, Matplotlib, and other libraries [25,26,27,28] of Python were used to develop the proposed and comparative models, respectively.

#### 2.2.2. Baseline Models

We compare the proposed model with the following baseline classifiers and feature selection methods:

Logistic regression (LR) [29] is a widely used statistical method for classification and regression task. LR is used when our target of interest has two possible dichotomy values that is limited to values between 0 and 1.

Random forest (RF) [30] is a parallel structured ensemble tree-based method that utilizes bagging to aggregate multiple decision tree classifiers. Each tree of the RF is trained on bootstrap samples of the training sets, using randomly selected features in the tree generation process; after that, each tree votes for the most popular class. In this study, we have configured the number of estimators in the random forest as 500, 750, 1000, 1250, 1500; moreover, quality of split-measured criteria was selected by “gini” for the Gini impurity and “entropy” for the information gain, respectively.

K-nearest neighbor (KNN) [31] is a supervised machine learning algorithm that can solve the classification task. In the classification phase, instances are classified to the class most frequently occurring amongst the neighbors, measured by the distance function. For the KNN classifier, hyperparameters of weights and number of neighbors were adjusted in this study. The weights set up to “uniform”, where all points in each neighborhood are weighted the same, or “distance” where closer points are more heavily weighted toward the decision. The setting of the neighbor numbers refers to how many neighboring points are to fall inside of one group. Furthermore, we have turned the value of the k number between 3 and 12.

Multilayer perceptron (MLP) and neural network (NN) [32,33]: MLP is the most typical type of neural network application using back propagation for training. Neural networks are inspired by composed of nodes. MLPs consist of at least three layers, such as input, hidden, and output. Nodes in neighboring layers are interconnected, but nodes in the same layer are not. Each connection between neurons is multiplied by the corresponding weight during training. Finally, the output of hidden nodes is estimated by applying an activation function and output layer makes decisions. For the MLP models, we use the one and three hidden layers with five nodes. NN models consist of turning the two to ten hidden layers using two to five nodes, respectively. These models are optimized by Adam. Moreover, we set the learning rate is 0.001 with “sigmoid” activation function.

Support vector machine recursive feature elimination (SVM-RFE) [34] estimate the weights of the features according to the support vectors, then eliminate the necessary features until the specified number of features is reached.

Random forest based feature selection (RFFS) [35] has been found to provide feature importance scores that are successfully utilized in data mining. On the other hand, RF classifier estimates the importance of each features, then naturally ranks them.

## 3. Experimental Results and Discussion

**Figure 2:**
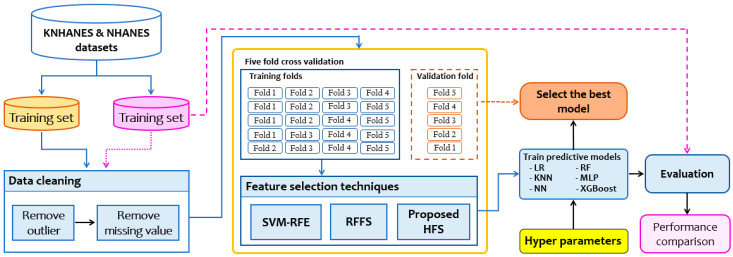
Design of comparative experiments for smoking-induced noncommunicable diseases prediction. K(NHANES): Korean (national health and nutrition examination survey); SVM-RFE: support vector machine recursive feature elimination; RFFS: random forest feature selection; HFS: hybrid feature selection; LR: logistic regression; KNN: k-nearest neighbors; NN: neural network; RF: random forest; MLP: multilayer perceptron; XGBoost: extreme gradient boosting.

The comparison of our proposed model with the baseline models is presented in this section. The flowchart of the experimental design is depicted in Figure 2. This study is reported according to the Transparent Reporting of a multivariable prediction model for Individual Prognosis or Diagnosis (TRIPOD) statement [36], shown in Appendix A (Table A1).

Initially, we preprocess the smoking-induced noncommunicable diseases (SiNCDs) datasets to eliminate missing values and outliers. Next, we elect a subset of representative features using feature selection methods. Finally, classifiers are used to build predictive models. Comparison findings would be to reveal a suitable combination of the feature selection methods and classifiers in an efficient predictive model among each dataset.

### 3.1. Dataset

In this study, the National Health and Nutrition Examination Survey datasets of South Korea (KNHANES) and the United States (NHANES) were used to build the proposed model and other existing baseline models for SiNCDs prediction.

KNHANES data is conducted by the Korea Centers for Disease Control and Prevention (KCDC) (http://knhanes.cdc.go.kr) [37]. It consists of a health examination of various numbers of diseases, health interviews, and nutrition surveys of the Korean population. NHANES is designed to assess the health and nutrition status of the general population in the United States. This nationwide survey is a major program of the National Center for Health Statistics (NCHS) that is part of the Centers for Disease Control and Prevention (CDC) (https://www.cdc.gov/nchs/nhanes) [38]. Generally, this survey examines approximately 5000 people each year across the United States. NHANES consists of demographic, socioeconomic, dietary, and health-related questions. Furthermore, some important features were surveyed from a minor of the population in both of the KNHANES and NHANES, similarly.

**Figure 3:**
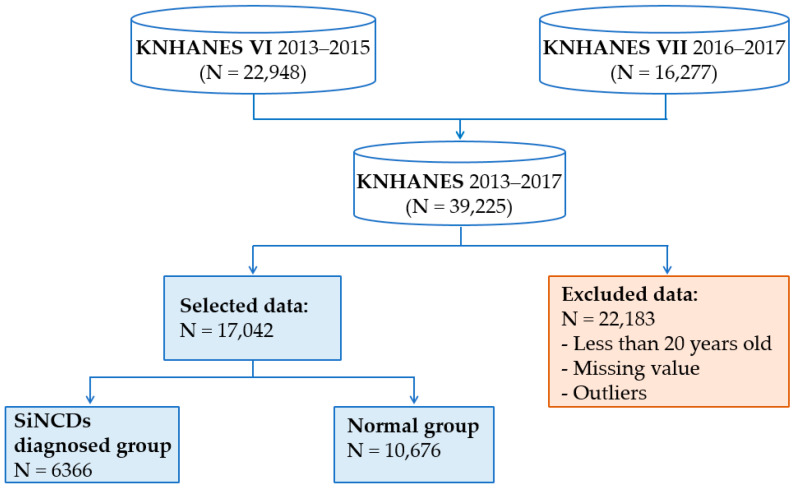
Sample selection procedure of the Korea National Health and Nutrition Examination Survey (KNHANES) dataset.

**Figure 4:**
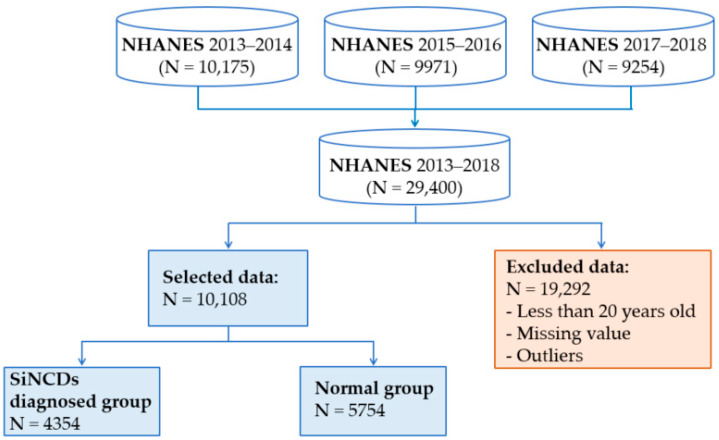
Sample selection procedure of National Health and Nutrition Examination Survey (NHANES) dataset.

We combined KNHANES datasets from 2013 through 2017, and NHANES datasets from 2013 through 2018, as shown in Figure 3 and Figure 4. Datasets contain a large number of missing values and outliers. It is well known that missing values reduce the statistical power and become a cause of the bias in the estimation of parameters. To prevent model complications, we excluded all of the missing values and outliers. The outliers were removed based on the interquartile range. Essentially 22,183 subjects of KNHANES and 19,292 subjects of NHANES were excluded due to missing value, outliers, and class targets, which were stored in given initial features. Additionally, subjects aged 20 years old were considered for our analysis for both KNHANES and NHANES datasets. This study was designed to include target and healthy control groups. Healthy control group was defined by subjects who had never smoked and had not been diagnosed with NCDs. On the other hand, target group was defined by subjects who had a history of one of the NCDs, for diabetes, prediabetes, asthma, heart failure, corona hearth disease, heart attack, stroke, hypertension, kidney failure, or angina, as well as had smoked at least 100 cigarettes in their life.

### 3.2. The Results of Hybrid Feature Selection (HFS)

At the first stage of HFS, *t*-test and chi-square statistical hypothesis tests are used to determine the null or alternative hypothesis in order to select significant features. The threshold of 0.01 indicates statistical significance. If the feature is of a numeric type, the significance is tested using *t*-test, otherwise, for the categorical features, chi-square is used. In the KNHANES dataset, the *p*-values of “self-management”, “daily activities”, and “economic activity status” features were estimated by 0.16, 0.58, and 0.50, respectively. While *p*-values of “Pulse regular or irregular?”, “Salt usage level”, and “Description of job/work situation” features were indicated by 0.19, 0.12, and 0.13, respectively. A large *p*-value (>0.01) indicates weak evidence against the null hypothesis; thus, we exclude these features due to criteria of the first stage of HFS.

Thereafter, we verify to check the collinearity between independent features using multicollinearity in regression analysis after eliminating the non-significant features in the second stage of HFS. Multicollinearity is one of the major concerns in causal inference. This second stage leads to prevent the causality issue that occurs when two or more features are highly correlated. It is challenging for a reliable estimation of the variable coefficients. It is suspected that multicollinearity will present if the variance inflation factor (VIF) lies between 5 and 10 in this study. If the VIF value is greater than those values, it investigates a high correlation among features that remain problematic.

In our analysis of the KNHANES dataset, we did not remove any features in terms of their low VIF values. On the contrary, we removed “annual household income” and “poor appetite or overeating” features that represented VIF values of 5.312 and 5.005 in the NHANES dataset. The detailed results of the bivariate and multicollinearity analysis of the KNHANES dataset in Table A2 and NHANES dataset in Table A3 are shown in Appendix B.

In the third stage of HFS, the least absolute shrinkage and selection operator (LASSO) helps to increase the prediction of the models by removing irrelevant features that are not related to target classes. The LASSO identified irrelevant features, such as “residence area”, “walk duration (hours)”, and “health checkup status” in KNHANES dataset, whereas, “ever told doctor had trouble sleeping?” and “number of healthcare counseling over the past year” in NHANES dataset. Thus, these irrelevant features were eliminated by assigning them a coefficient equal to zero. In terms of the three-stage HFS method, we selected sufficiently representative 26 of 32 features in KNHANES and 28 of 35 features in NHANES. Moreover, these are used as inputs to the predictive model.

### 3.3. The Results of the Comparative Analysis

To prove the efficient XGBoost based framework equipped with the HFS method for SiNCDs, it is compared with other current techniques, including LR, RF, KNN, MLP, NN, and XGBoost incorporated with HFS, SVM-RFE, and RFFS methods in terms of KNHANES and NHANES datasets.

In this study, the entire process of parameter estimation in most baseline models are utilized based on the research paper [39]. For evaluating the prediction models, we split the data into 80% for the training set and 20% for the evaluation set. In order to prevent overfitting, a 5-fold cross-validation procedure [40] is applied to the training set. In the procedure of 5-fold cross-validation, the dataset is randomly partitioned into five parts: 4 folds of the training set are used to train classification models, and the remaining 1 fold is used to validate the model. To evaluate the performance of the predictive models, the classification accuracy, sensitivity, specificity, precision, f-score, and area under the receiver operating characteristic curve (AUC) [41,42] were used.

Table 2 shows the performances of all predictive models in the KNHANES dataset and highest performance of evaluation metrics are marked in bold. For KNHANES dataset, XGBoost with HFS model achieved the highest accuracy of 0.8812 precision of 0.8737 and F-score of 0.8707. NN with RFFS models outperformed the best sensitivity of 0.8871 and specificity of 0.8902. Following by it, the second-best accuracy of 0.8758 and precision of 0.8691 were achieved by NN with HFS model, a specificity of 0.8496 is reached by RF with HFS, and sensitivity of 0.8782 and F-score of 0.8703 was reached by XGBoost with RFFS in the prediction of SiNCDs. As can be seen, KNN with SVM-RFE based predictive model performed slightly lower results compared with other predictive models in terms of the evaluation metrics.

As shown in Table 3, we have summarized results of the predictive model in the NHANES dataset and highest performance of evaluation metrics are marked in bold. For the NHANES dataset, the best model was distinguished by our proposed XGBoost with HFS in terms of the accuracy, sensitivity, specificity, precision, and F-score, which reached 0.9309, 0.8944, 0.9522, 0.8874, and 0.8909, respectively. Moreover, second-best performances were yielded by the XGBoost with RFFS, which achieved the accuracy of 0.9029, sensitivity of 0.8507, specificity of 0.9379, precision of 0.8264, and f-score of 0.8384. Otherwise, it can be seen that the XGBoost classifier exhibited the best capability of probability prediction results incorporating different feature selection methods, significantly.

On the contrary, SVM-RFE method achieved the lowest prediction performances compared with the other feature selection method; therefore, that SVM-RFE method is not suitable for SiNCDs predictive models. Besides, the RFFS method performed computable results with the proposed HFS method in our prediction task. It is well known that accuracy is the most appropriate metric for evaluating predictive models.

**Figure 5:**
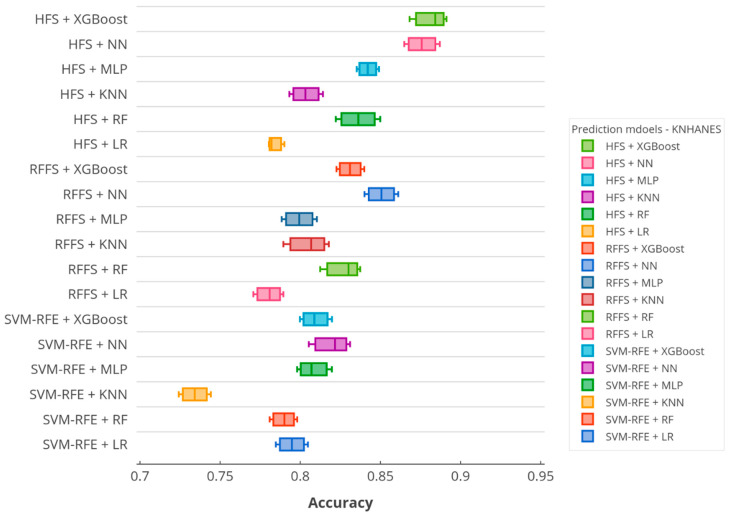
Boxplot of the accuracy over prediction models in the KNHANES dataset.

**Figure 6:**
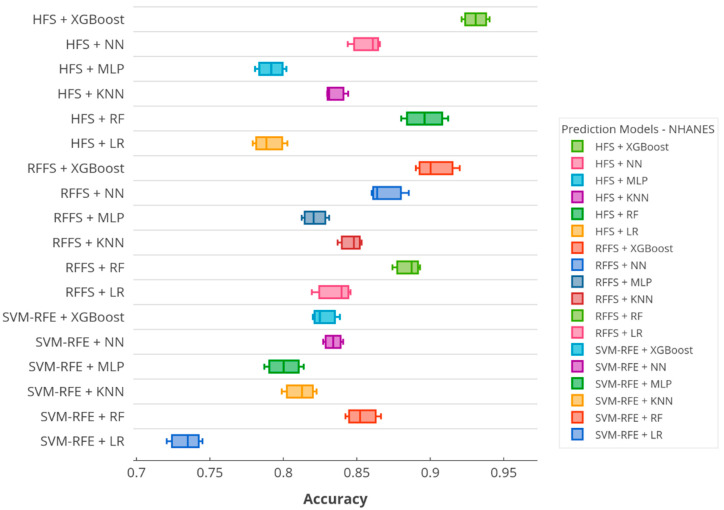
Boxplot of the accuracy over prediction models in the NHANES dataset.

According to the accuracy score, Figure 5 and Figure 6 illustrate the boxplots of the prediction models in the KNHANES and NHANES datasets. In the figures, *x*-axis denotes the accuracy scores and *y*-axis presents the utilized predictive models on SiNCDs. As depicted in Figure 5, the proposed XGBoost based framework equipped with HFS presented the highest score in the KNHANES dataset. Thereafter, NN with HFS showed the second-highest score, otherwise, the HFS method was capable of predicting the target in this task. By contrast, KNN with SVM-RFE and RFFS with RF models reached the worst scores of 0.7342 and 0.7804, respectively. Furthermore, the proposed XGBoost with the HFS model also achieved the highest score in the NHANES dataset as represented in Figure 6. Interestingly, the worst scores of 0.7349 and 0.7903 were exhibited by the LR with SVM-RFE and LR with HFS, respectively. In the NHANES dataset, the LR baseline classifier determined the worst predictive model, but results slightly improved when this classifier was combined with RFFS.

Table 4 shows the AUC analysis results of the predictive models in the KNHANES and NHANES datasets; results were verified by statistical significance test. It is evidently seen that, in terms of AUC, predictive models were statistically significant. A careful look at the results of KNHANES dataset, the highest performance of 0.8887 (95% CI, 0.8659–0.9005) was scored by NN with RFFS, following by 0.8402 (95% CI, 0.8384–0.8635) was achieved by our proposed XGBoost with the HFS model. Whilst SVM-RFE with the KNN model performed the lowest performance of 0.7170 (95% CI, 0.7094–0.7390) in this analysis. For the NHANES dataset, the proposed model indicated the better performances of 0.9233 (95% CI, 0.9073–0.9345) than other benchmark baselines. Moreover, XGBoost with the RFFS model achieved the second-best performance of 0.8943 (95% CI, 0.8757–0.9013), followed by HFS with RF of 0.8647 (95% CI, 0.8564–0.8859), significantly. These results provided evidence that our ensemble models evaluated the better performances among other baseline models in the NHANES dataset.

**Figure 7:**
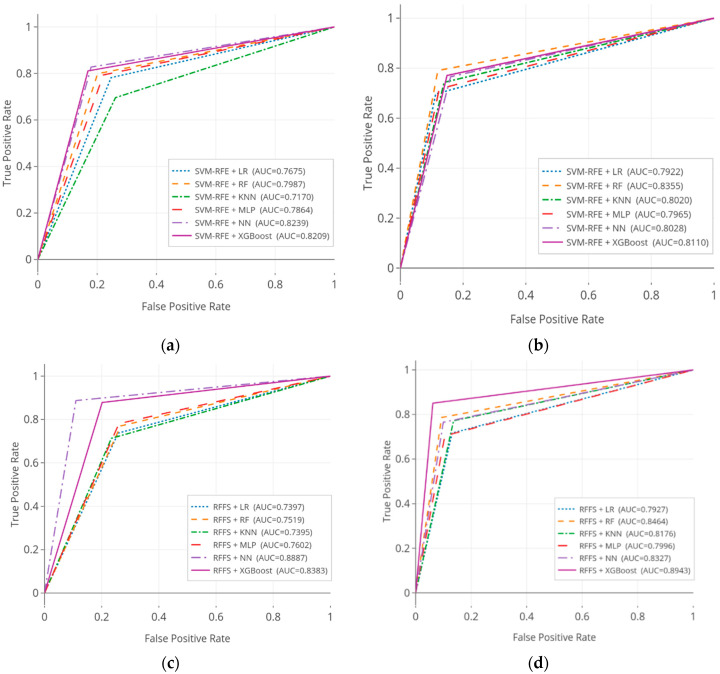

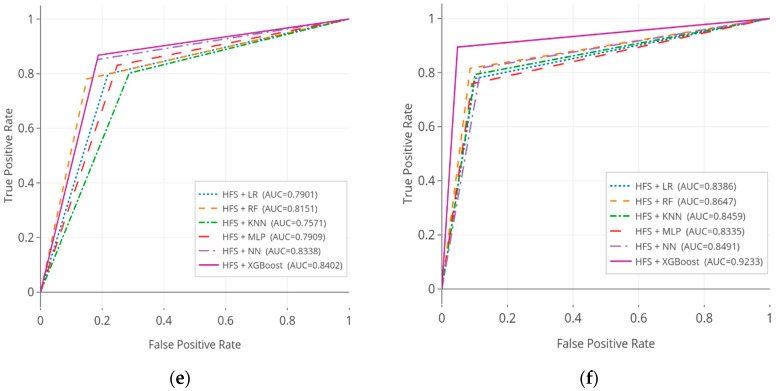
The comparison of receiver operating characteristic (ROC) curves for smoking-induced noncommunicable diseases (SiNCDs) prediction in the KNHANES and NHANES datasets. (**a**) ROC of SVM-RFE based models in KNHANES; (**b**) ROC of SVM-RFE based models in NHANES; (**c**) ROC of RFFS based models in KNHANES; (**d**) ROC of RFFS based models in NHANES; (**e**) ROC of HFS based models in KNHANES; (**f**) ROC of HFS based models in NHANES.

Figure 7 illustrates the ROC curves for the SiNCDs predictive models on the three kinds of feature selection methods across KNHANES and NHANES datasets. In ROC curves analysis, we can demonstrate the separation and discrimination ability of the predictive models. The ROC curve was plotted with the measurements of true positive rate (sensitivity) along with the *y*-axis, and false positive (1-specificity) along with the *x*-axis. For the KNHANES dataset, ROC curves of NN and XGBoost classifiers represented high results across SVM-RFE based models. RFFS based NN and XGBoost models indicated notably higher results compared with RFFS based models, significantly. Moreover, the three-stage HFS method based classifiers showed fluently good results among other baselines. ROC curve analysis of the NHANES dataset, the RF, and XGBoost classifiers incorporated with SVM-RFE had higher results across other SVM-RFE based models. Moreover, it can be seen that the proposed HFS based XGBoost model emerged as being a good combination model, as it can reach notable significant results than the other HFS and RFFS based baseline models.

The results of the KNHANES dataset reported in Table 4 and Figure 6 indicate the enhanced performances of not only the XGBoost classifier, but also the RFFS based NN considered as a computable model during the comparison task.

### 3.4. Interpretability of Predictive Model

Prediction performance concerns the ability of the best predictive model to make correct decisions. Meanwhile, predictive model interpretability concerns the understanding of model decisions by humans. Interpretability methods can be categorized into three types: explain data, build an inherently interpretable model (in modeling), and allow to explain it after building the models [43]. In practice, there have been some needs for using machine learning models to ensure which factors are used to make key decisions with boosted trees [44]. Model inherent interpretability is important to get a reasoning behind the predictive models. However, model interpretability tends to be ignored in previous studies [15,16].

**Figure 8:**
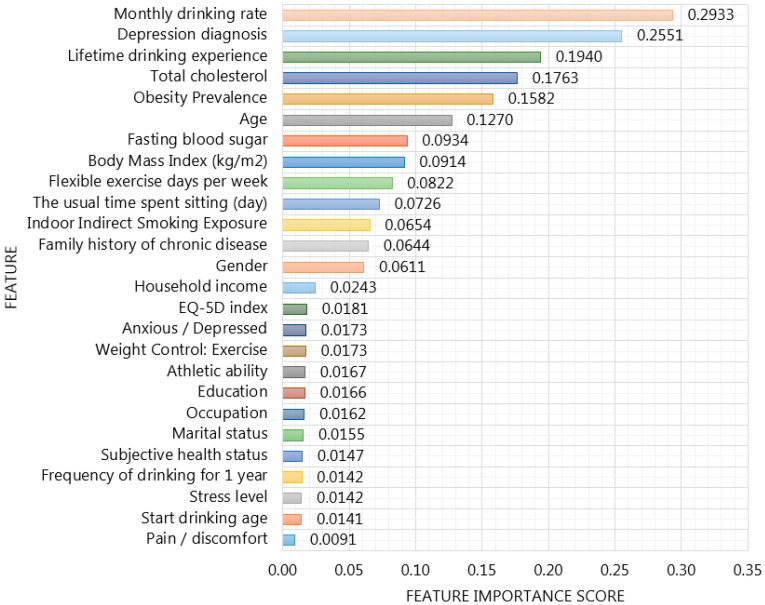
Feature Importance of XGBoost based framework incorporated with hybrid feature selection in the KNHANES dataset.

**Figure 9:**
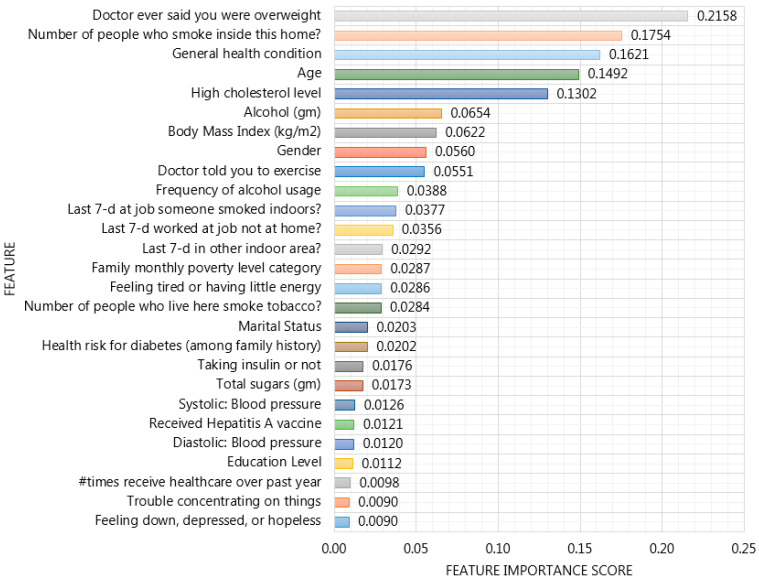
Feature Importance of XGBoost based framework incorporated with hybrid feature selection in the NHANES dataset.

Our proposed XGBoost based framework incorporated with HFS provides important features in order to enhance the interpretability of SiNCDs prediction model across KNHANES and NHANES datasets as depicted in Figure 8 and Figure 9. To ensure model interpretability, features were sorted in descending order of their importance scores in XGBoost based model construction in each dataset. For the KNHANES dataset, “monthly drinking rate”, “depression diagnosis”, “lifetime drinking experience”, and “total cholesterol“ were maintained as the most useful features, with importance scores of 0.2933, 0.2551, 0.1940, and 0.1763 to predict SiNCDs among the Korean population, as shown in Figure 8. While with the NHANES dataset analysis, it is evidently shown that “doctor ever said you were overweight”, “the number of people who smoke inside this home”, “general health condition”, and “age” were determined as the most important scores with 0.2158, 0.1754, 0.1621, and 0.1492, respectively, across the general population in the United States, as presented in Figure 9.

Accordingly, alcohol drinking frequency and consumption mostly occurred as highly scored features in the Korean population. Moreover, studies [45,46] found a similar factor for NCDs across Thailand and Korea, although, SiNCDs of Korea and the United States were eminently caused by overweight, cholesterol levels, and obesity, similarly. This result is similar to that study [47], where high rates of overweight and obesity increased the burden of type 2 diabetes, coronary heart disease, and stroke in most countries in the Middle East. According to Kinra et al. [48], the risks for severe illness from NCDs increased with older adults in 1600 villages from 18 states in India. Moreover, they identified that lower socioeconomic status is associated with smoking, alcohol use, low intake of fruit (vegetables), and underweight, whereas, higher socioeconomic status is associated with greater exposure to obesity, dyslipidemia, diabetes in men, and hypertension in women. In particular, authors highlighted that the prevalence of cigarette smoking among men and obesity among women was significantly higher in rural India. Dan et al. [49] examined the main (and interaction) effects of age, gender, body mass index (BMI), and dietary intake among Korean hypertensive patients. Their analysis has found that BMI, energy intake, and cholesterol intake decreased in the older-aged group compared to the middle-aged group. In addition, their findings interpreted that both genders considered weight and dietary management for reducing the incidence of hypertension. In one study, Maimela et al. [50] determined the prevalence of risk factors of NCDs among rural communities in the Limpopo Province of South Africa. Their results defined that tobacco prevalence, alcohol consumption, and being overweight has consistently higher association with NCDs among adults.

Otherwise, most of the notable risk factors for NCDs in each dataset were represented as modifiable. It is well known that modifiable risk factors are behaviors and exposures that are highly associated with the risk of developing various diseases. In order to prevent and correct these modifiable risk factors, it required actions, such as smoking cessation, alcohol reduction, and exercise in public health. The highly scored features enhance rational decisions in smoking-related health concerns and should be collected in SiNCDs prediction data. In addition, this analysis is expected to compare the similarities and differences between populations of two different countries for SiNCDs prediction.

## 4. Conclusions

In this study, we proposed extreme gradient boosting (XGBoost) based framework incorporated with a hybrid feature selection (HFS) method for SiNCDs prediction, using real-world National Health and Nutrition Examination Survey (NHANES) datasets of South Korea and the United States. The proposed framework consisted of three main steps: first, a three-stage hybrid feature selection method to select important features, then built the XGBoost predictive model to accomplish the task of predicting SiNCDs, and, finally, the framework provided the XGBoost based feature importance scores to enhance the understanding of the reasoning behind the predictive models. The model under the proposed framework was compared against various existing baselines and it has shown superior performance in terms of accuracy measures across each dataset. We also determined the most representative features for SiNCDs in the general populations of South Korea and the United States. Although the study has successfully demonstrated that smoking induced serious health hazards, it has certain limitations in terms of interpretability of deep learning, known as the black-box. In the future, this study can be extended by addressing the problem of global and local interpretability of black-box models and causal effect in the scenario of predictive models.

## Figures and Tables

**Table 1:** Searching space of XGBoost model.

Parameters	Symbol	Search Space
Maximum tree depth	$D_{max}$	2, 4, 6, 8
Minimum child weight	$w_{mc}$	2, 3, 4, 5
Early stop round	e	100
Learning rate	τ	0.1
Number of boost	N	60
Maximum delta step	δ	0.4, 0.6, 0.8, 1
Subsample ratio	$r_{s}$	0.9, 0.95, 1
Column subsample ratio	$r_{c}$	0.9, 0.95, 1
Gamma	γ	0, 0.001

**Table 2:** Evaluation results of the prediction models in the Korea National Health and Nutrition Examination Survey dataset.

Feature Selection	Classifier	Accuracy	Sensitivity	Specificity	Precision	F-Score
SVM-RFE	LR	0.7948	0.7818	0.7532	0.7676	0.7746
	RF	0.7890	0.7989	0.7984	0.8115	0.8052
	KNN	0.7342	0.6958	0.7381	0.7961	0.7426
	MLP	0.8070	0.7936	0.7791	0.8016	0.7976
	NN	0.8197	0.8274	0.8203	0.8387	0.8330
	XGBoost	0.8098	0.8108	0.8310	0.8533	0.8315
RFFS	LR	0.7804	0.7371	0.7422	0.8024	0.7684
	RF	0.8264	0.7699	0.7338	0.8236	0.7958
	KNN	0.8048	0.7128	0.7661	0.7753	0.7427
	MLP	0.7994	0.7808	0.7396	0.8115	0.7959
	NN	0.8507	**0.8871**	**0.8902**	0.8522	0.8693
	XGBoost	0.8311	0.8782	0.7984	0.8626	0.8703
HFS	LR	0.7834	0.7989	0.7813	0.7959	0.7974
	RF	0.8362	0.7805	0.8496	0.8115	0.7957
	KNN	0.8032	0.8018	0.7123	0.7872	0.7944
	MLP	0.8421	0.8305	0.7513	0.8257	0.8281
	NN	0.8758	0.8518	0.8158	0.8691	0.8604
	XGBoost	**0.8812**	0.8677	0.8126	**0.8737**	**0.8707**

SVM-RFE: support vector machine recursive feature elimination; RFFS: random forest feature selection; HFS: hybrid feature selection; LR: logistic regression; KNN: k-nearest neighbors; NN: neural network; RF: random forest; MLP: multilayer perceptron; XGBoost: extreme gradient boosting. Highest scores are marked in bold.

**Table 3:** Evaluation results of the prediction models in the National Health and Nutrition Examination Survey dataset.

Feature Selection	Classifier	Accuracy	Sensitivity	Specificity	Precision	F-Score
SVM-RFE	LR	0.7349	0.6969	0.8874	0.7086	0.7027
	RF	0.8522	0.7904	0.8805	0.8157	0.8029
	KNN	0.8118	0.7432	0.8608	0.8105	0.7754
	MLP	0.8002	0.7171	0.8759	0.6816	0.6989
	NN	0.8339	0.7659	0.8397	0.7609	0.7634
	XGBoost	0.8248	0.7707	0.8512	0.8066	0.7882
RFFS	LR	0.8356	0.7169	0.8685	0.6938	0.7052
	RF	0.8741	0.7863	0.9065	0.7356	0.7601
	KNN	0.8444	0.7716	0.8635	0.7594	0.7655
	MLP	0.8221	0.7043	0.8949	0.6842	0.6941
	NN	0.8639	0.7651	0.9003	0.7534	0.7592
	XGBoost	0.9029	0.8507	0.9379	0.8264	0.8384
HFS	LR	0.7903	0.7781	0.8990	0.7732	0.7756
	RF	0.8961	0.8157	0.9136	0.7857	0.8004
	KNN	0.8363	0.7928	0.8990	0.7981	0.7954
	MLP	0.7918	0.7586	0.9083	0.7635	0.7610
	NN	0.8553	0.8173	0.8808	0.7934	0.8052
	XGBoost	**0.9309**	**0.8944**	**0.9522**	**0.8874**	**0.8909**

Highest scores are marked in bold.

**Table 4:** Statistical significance test of the area under the curve results for predictive models in the KNHANES and NHANES datasets.

Feature Selection	Classifier	KNHANES Dataset			NHANES Dataset		
		AUC	CI 95%	*p*-Value	AUC	CI 95%	*p*-Value
SVM-RFE	LR	0.7675	0.7474–0.7896	<0.001	0.7922	0.7731–0.8088	<0.001
	RF	0.7987	0.7869–0.8118	<0.001	0.8355	0.8254–0.8668	<0.001
	KNN	0.7170	0.7094–0.7390	<0.001	0.8020	0.7818–0.8210	<0.001
	MLP	0.7864	0.7703–0.8001	<0.001	0.7965	0.7851–0.8180	<0.001
	NN	0.8239	0.8017–0.8405	<0.001	0.8028	0.7981–0.8447	<0.001
	XGBoost	0.8209	0.8097–0.8327	<0.001	0.8110	0.8041–0.8315	<0.001
RFFS	LR	0.7397	0.7713–0.7971	<0.001	0.7927	0.7806–0.8197	<0.001
	RF	0.7519	0.7683–0.8111	<0.001	0.8464	0.8359–0.8637	<0.001
	KNN	0.7395	0.7570–0.8037	<0.001	0.8176	0.8070–0.8308	<0.001
	MLP	0.7602	0.7721–0.8267	<0.001	0.7996	0.7872–0.8135	<0.001
	NN	**0.8887**	0.8659–0.9005	<0.001	0.8327	0.8206–0.8492	<0.001
	XGBoost	0.8383	0.8245–0.8567	<0.001	0.8943	0.8757–0.9013	<0.001
HFS	LR	0.7901	0.7812–0.8253	<0.001	0.8386	0.8234–0.8539	<0.001
	RF	0.8151	0.7947–0.8286	<0.001	0.8647	0.8564–0.8859	<0.001
	KNN	0.7571	0.7401–0.7796	<0.001	0.8459	0.8284–0.8653	<0.001
	MLP	0.7909	0.7846–0.8243	<0.001	0.8335	0.8195–0.8506	<0.001
	NN	0.8338	0.8249–0.8494	<0.001	0.8491	0.8310–0.8588	<0.001
	XGBoost	0.8402	0.8384–0.8635	<0.001	**0.9233**	0.9073–0.9345	<0.001

Highest scores are marked in bold.

## Appendix A

**Table A1:** Transparent Reporting of a multivariable prediction model for Individual Prognosis or Diagnosis (TRIPOD) Checklist: Prediction Model Development and Validation.

Section/Topic	Item		Checklist Item	Page
**Title and abstract**				
Title	1	D; V	Identify the study as developing and/or validating a multivariable prediction model, the target population, and the outcome to be predicted.	1
Abstract	2	D; V	Provide a summary of objectives, study design, setting, participants, sample size, predictors, outcome, statistical analysis, results, and conclusions.	2
**Introduction**				
Background and objectives	3a	D; V	Explain the medical context (including whether diagnostic or prognostic) and rationale for developing or validating the multivariable prediction model, including references to existing models.	2–3
	3b	D; V	Specify the objectives, including whether the study describes the development or validation of the model or both.	3–5
**Methods**				
Source of data	4a	D; V	Describe the study design or source of data (e.g., randomized trial, cohort, or registry data), separately for the development and validation datasets, if applicable.	6–7
	4b	D; V	Specify the key study dates, including start of accrual; end of accrual; and, if applicable, end of follow-up.	6–7
Participants	5a	D; V	Specify key elements of the study setting (e.g., primary care, secondary care, general population), including number and location of centers.	6–7
	5b	D; V	Describe eligibility criteria for participants.	6–7 Figure 3 and Figure 4
	5c	D; V	Give details of treatments received, if relevant.	n/a
Outcome	6a	D; V	Clearly define the outcome that is predicted by the prediction model, including how and when assessed.	8–12
	6b	D; V	Report any actions to blind assessment of the outcome to be predicted.	9–12
Predictors	7a	D; V	Clearly define all predictors used in developing the multivariable prediction model, including how and when they were measured.	7–8
	7b	D; V	Report any actions to blind assessment of predictors for the outcome and other predictors.	7–8
Sample size	8	D; V	Explain how the study size was arrived at.	7–8
Missing data	9	D; V	Describe how missing data were handled (e.g., complete-case analysis, single imputation, multiple imputation) with details of any imputation method.	7
Statistical analysis methods	10a	D	Describe how predictors were handled in the analyses.	7–8
	10b	D	Specify type of model, all model-building procedures (including any predictor selection), and method for internal validation.	4–5, 8 Figure 1
	10c	V	For validation, describe how the predictions were calculated.	8
	10d	D; V	Specify all measures used to assess model performance and, if relevant, to compare multiple models.	8 Figure 2
	10e	V	Describe any model updating (e.g., recalibration) arising from the validation, if done.	8
Risk groups	11	D; V	Provide details on how risk groups were created, if done.	7
Development vs. validation	12	V	For validation, identify any differences from the development data in setting, eligibility criteria, outcome, and predictors.	8
**Results**				
Participants	13a	D; V	Describe the flow of participants through the study, including the number of participants with and without the outcome and, if applicable, a summary of the follow-up time. A diagram may be helpful.	Figure 3 and Figure 4
	13b	D; V	Describe the characteristics of the participants (basic demographics, clinical features, available predictors), including the number of participants with missing data for predictors and outcome.	Figure 3 and Figure 4
	13c	V	For validation, show a comparison with the development data of the distribution of important variables (demographics, predictors, and outcome).	Table A2 and Table A3
Model development	14a	D	Specify the number of participants and outcome events in each analysis.	7–8
	14b	D	If done, report the unadjusted association between each candidate predictor and outcome.	8–9
Model specification	15a	D	Present the full prediction model to allow predictions for individuals (i.e., all regression coefficients, and model intercept or baseline survival at a given time point).	10
	15b	D	Explain how to use the prediction model.	8–12
Model performance	16	D; V	Report performance measures (with CIs) for the prediction model.	11 Table 4
Model-updating	17	V	If done, report the results from any model updating (i.e., model specification, model performance).	12–13
**Discussion**				
Limitations	18	D; V	Discuss any limitations of the study (such as non-representative sample, few events per predictor, missing data).	15
Interpretation	19a	V	For validation, discuss the results with reference to performance in the development data, and any other validation data.	13–14
	19b	D; V	Give an overall interpretation of the results, considering objectives, limitations, results from similar studies, and other relevant evidence.	14–15
Implications	20	D; V	Discuss the potential clinical use of the model and implications for future research.	14–15
**Other information**				
Supplementary information	21	D; V	Provide information about the availability of supplementary resources, such as study protocol, web calculator, and datasets.	16–18
Funding	22	D; V	Give the source of funding and the role of the funders for the present study.	15

Items relevant only to the development of a prediction model are denoted by D, items relating solely to a validation of a prediction model are denoted by V, and items relating to both are denoted D; V.

## Appendix B

**Table A2:** Bivariate and Multicollinearity analysis for KNHANES dataset.

	Features	*p*-Value	Multicollinearity Coefficient
1	Gender	<0.01	1.081
2	Age	<0.01	1.523
3	Household income	<0.01	2.901
4	Education	<0.01	1.125
5	Occupation	<0.01	2.016
6	Marital status	<0.01	3.553
7	Subjective health status	<0.01	1.778
8	Depression diagnosis	<0.01	1.286
9	Health checkup status	<0.01	1.047
10	Athletic ability	<0.01	1.124
11	Self-management	0.16	~
12	Daily activities	0.58	~
13	Pain/discomfort	<0.01	2.229
14	Anxious/Depressed	<0.01	4.345
15	EQ-5D index	<0.01	2.473
16	Economic activity status	0.50	~
17	Weight control: exercise	<0.01	3.329
18	Lifetime drinking experience	<0.01	1.171
19	Start drinking age	<0.01	1.003
20	Frequency of drinking for 1 year	<0.01	1.532
21	Monthly drinking rate	<0.01	3.152
22	Stress level	<0.01	3.033
23	Indoor indirect smoking exposure	<0.01	1.221
24	The usual time spent sitting (day)	<0.01	1.096
25	Walk duration (hours)	<0.01	1.114
26	Family history of chronic disease	<0.01	1.087
27	Body mass index (kg/m^2^)	<0.01	1.048
28	Obesity prevalence	<0.01	2.675
29	Fasting blood sugar	<0.01	2.536
30	Total cholesterol	<0.01	1.151
31	Flexible exercise days per week	<0.01	1.038
32	Residence area	<0.01	1.547

**Table A3:** Bivariate and Multicollinearity analysis for NHANES dataset.

	Features	*p*-Value	Multicollinearity Coefficient
1	Gender	<0.01	2.005
2	Age	<0.01	1.008
3	Body mass index (kg/m^2^)	<0.01	3.005
4	Pulse regular or irregular?	0.19	~
5	Systolic: blood pressure	<0.01	2.015
6	Diastolic: blood pressure	<0.01	1.875
7	Education level	<0.01	1.076
8	Marital status	<0.01	3.092
9	Total number of people in the household	0.32	~
10	Annual household income	<0.01	5.312
11	Health risk for diabetes (among family history)	<0.01	3.533
12	Taking insulin or not	<0.01	1.453
13	Number of healthcare counseling over past year	<0.01	2.027
14	Salt usage level	0.12	~
15	Total sugars (gm)	<0.01	1.298
16	Alcohol (gm)	<0.01	2.479
17	Frequency of alcohol usage	<0.01	2.204
18	High cholesterol level	<0.01	1.340
19	General health condition	<0.01	1.199
20	#times receive healthcare over past year	<0.01	1.249
21	Received hepatitis A vaccine	<0.01	2.012
22	Family monthly poverty level category	<0.01	1.004
23	Doctor ever said you were overweight	<0.01	1.012
24	Doctor told you to exercise	<0.01	1.004
25	Feeling down, depressed, or hopeless	<0.01	1.012
26	Feeling tired or having little energy	<0.01	1.004
27	Poor appetite or overeating	<0.01	5.005
28	Trouble concentrating on things	<0.01	2.292
29	Description of job/work situation	0.13	~
30	Ever told doctor had trouble sleeping?	<0.01	1.012
31	Number of people who live here smoke tobacco?	<0.01	1.035
32	Number of people who smoke inside this home?	<0.01	1.424
33	Last 7-d worked at job not at home?	<0.01	2.404
34	Last 7-d at job someone smoked indoors?	<0.01	1.205
35	Last 7-d in other indoor area?	<0.01	2.108
